# Effects of Stress and MDMA on Hippocampal Gene Expression

**DOI:** 10.1155/2014/141396

**Published:** 2014-01-09

**Authors:** Georg F. Weber, Bethann N. Johnson, Bryan K. Yamamoto, Gary A. Gudelsky

**Affiliations:** ^1^James Winkle College of Pharmacy, University of Cincinnati, 3225 Eden Avenue, Cincinnati, OH 45267, USA; ^2^Department of Neurosciences, University of Toledo School of Medicine, Toledo, OH 43606, USA

## Abstract

MDMA (3,4-methylenedioxymethamphetamine) is a substituted amphetamine and popular drug of abuse. Its mood-enhancing short-term effects may prompt its consumption under stress. Clinical studies indicate that MDMA treatment may mitigate the symptoms of stress disorders such as posttraumatic stress syndrome (PTSD). On the other hand, repeated administration of MDMA results in persistent deficits in markers of serotonergic (5-HT) nerve terminals that have been viewed as indicative of 5-HT neurotoxicity. Exposure to chronic stress has been shown to augment MDMA-induced 5-HT neurotoxicity. Here, we examine the transcriptional responses in the hippocampus to MDMA treatment of control rats and rats exposed to chronic stress. MDMA altered the expression of genes that regulate unfolded protein binding, protein folding, calmodulin-dependent protein kinase activity, and neuropeptide signaling. In stressed rats, the gene expression profile in response to MDMA was altered to affect sensory processing and responses to tissue damage in nerve sheaths. Subsequent treatment with MDMA also markedly altered the genetic responses to stress such that the stress-induced downregulation of genes related to the circadian rhythm was reversed. The data support the view that MDMA-induced transcriptional responses accompany the persistent effects of this drug on neuronal structure/function. In addition, MDMA treatment alters the stress-induced transcriptional signature.

## 1. Introduction

3,4-Methylenedioxymethamphetamine (MDMA) is a ring-substituted amphetamine analog. The primary effect of this drug is to alter perception, cognition, or mood. People who take it recreationally generally experience a feeling of elation [1]. Its short-term euphoria-inducing properties have made MDMA (ecstasy) a popular drug of abuse. These mood altering effects may also prompt MDMA intake by individuals who desire to overcome stressful experiences. In particular, MDMA treatment is under study to mitigate the clinical symptoms of PTSD [2]. However, it is not known whether or how the effects of MDMA may be altered in the context of chronic stress. It is conceivable that the pathophysiologic changes induced by stress could alter the efficacy or increase the toxicity of MDMA.

The hippocampus is a brain area that may be particularly susceptible to the effects of both MDMA and chronic stress. Chronic unpredictable stress suppresses neurogenesis within the hippocampus [3] and reduces dendritic complexity and spine density in this brain region [4, 5]. Chronic stress also increases the vulnerability of hippocampal neurons to damage produced by neurotoxins and drugs [6, 7]. The repeated administration of MDMA produces well-documented, persistent deficits in biochemical markers of 5-HT axon terminals in multiple brain regions, including the hippocampus, in rodents and humans [8–11]. These findings have been viewed as evidence of MDMA-induced distal axotomy of 5-HT neurons [12]. Moreover, exposure to chronic unpredictable stress potentiates MDMA-induced 5-HT neurotoxicity in the hippocampus [13].

Beyond the persistent effects of MDMA on 5-HT axon terminals, there is evidence that MDMA produces neuronal degeneration within multiple brain regions, including the hippocampus [14–17]. Although the identity of the targeted neuronal populations has not been determined, Anneken et al. [18] and Perrine et al. [19] have reported that MDMA reduces biochemical markers of hippocampal GABAergic neurons.

The hippocampus is known to be critical for learning and memory function [20]. Deficits in spatial learning and memory [21–24] and impairments in developing a conditioned place preference to sex [25] have been reported in rats treated with MDMA. In human abusers of MDMA, the most consistent finding is that of impairments in short-term memory, particularly verbal recall [26, 27], as well as deficits in tasks that assess executive functioning [28, 29].

The purpose of the present study was to investigate the interaction between chronic stress and MDMA treatment within the hippocampus at the molecular level, as evaluated from transcriptional responses quantified by microarray analysis.

## 2. Materials and Methods

### 2.1. Animals and Stress Exposure

Adult, male rats (250–300 g) of the Sprague-Dawley strain (Harlan Laboratories, Indianapolis, IN) were used in this study. Animals had free access to food and water in a temperature- and humidity-controlled room. All procedures were performed in adherence to the NIH guidelines and were approved by the institutional animal care and use committee.

Two groups of rats were exposed to chronic, unpredictable stress that consisted of various types, times, and durations of stressors. This regimen has been described previously [30] and consisted of the following:  day 1: 10:00 a.m. 50-min exposure to cold (4°C) and 1:00 p.m. 60 min of restraint; day 2: 11:00 a.m. 60 min of cage agitation and 6:00 p.m. lights on overnight; day 3: 10:00 a.m. 3 hr of lights off and 3:00 p.m. 3 min of swim stress; day 4: 11:00 a.m. 50 min of restraint and 5:00 p.m. food and water deprivation overnight; day 5: 12:00 p.m. 15 min cold room isolation and 12:30 p.m. isolation housing overnight; day 6: 10:00 a.m. 4 min of swim stress and 6:00 p.m. lights on overnight; day 7: 9:00 a.m. 2 hr of lights off and 6 : 00 p.m. food and water deprivation overnight; day 8: 10:00 a.m. 30 min of restraint and 3:00 p.m. 40 min of cage agitation; day 9: 11:00 a.m. 3 min swim stress and 6:00 p.m. lights on overnight; day 10: 10:00 a.m. 3 hr of lights off and 1:00 p.m. 20 min of cage agitation. Two groups of nonstressed rats were transported daily to the area of stressors but were not exposed to the stressors. After completion of each stressor, the rats were returned to the housing area.

### 2.2. Drug Treatment

Control and stress-exposed rats were injected with racemic 3,4-methylenedioxymethamphetamine (MDMA) (generously provided by the National Institute on Drug Abuse) or vehicle (0.15 N NaCl) 24 hr following exposure to the last stressor. The rats received MDMA (10 mg/kg, ip) or vehicle at 2 hr intervals for a total of 4 injections. The rats were euthanized for removal of the hippocampus 24 hr following the first injection of MDMA or vehicle.

### 2.3. RNA Extraction and Hybridization

RNA was extracted from the hippocampi with 1.5 mL TriReagent according to the instructions by the manufacturer. The RNA samples were quantified by UV absorbance (260 and 280 nm) and their integrity was confirmed by Agilent Bioanalyzer 2100. Intact total RNA amounts of at least 50 ng were amplified using the NuGen Applause WT-Amp Plus ST system (http://www.nugeninc.com/nugen/index.cfm/products/apl/ applause-rna-amplification-systems/). The samples were then biotinylated with the Ambion Biotin-Enhanced Message Amp II kit. The GeneChip Rat Gene 1.0 ST Array was hybridized with 2.5 *μ*g of fragmented biotinylated aRNA. The hybridization, staining, and washing were carried out using the Affymetrix GeneChip Hybridization Wash and Stain Kit following the manufacturer's protocols. Briefly, the arrays were hybridized for 16 hr at 45°C, followed by protocol FS450_0007 for staining and washing the GeneChips in the Affymetrix Fluidics Station 450. The GeneChips were scanned with Affymetrix GeneChip Scanner 3000 7 G using GCOS and software and preset settings. The Rat Gene 1.0 ST chip provides comprehensive coverage of the transcribed rat genome by analyzing over 27,000 protein coding transcripts, over 24,000 Entrez genes, and alternatively spliced transcript variants with probes designed to maximize coverage of exons. For each treatment group, there were 4-5 hybridizations.

### 2.4. Microarray Analysis

The data were analyzed to identify differentially expressed genes between the following conditions: (1) no stress saline versus stress saline, (2) no stress MDMA versus stress MDMA, (3) no stress saline versus no stress MDMA, and (4) stress saline versus stress MDMA. Four and five biological replicate arrays for Saline and MDMA samples, respectively, were performed.

Once the arrays had been scanned and the data files were generated, the results were analyzed by the UC Laboratory for Statistical Genomics and Systems Biology. Analysis was performed using statistical software R and the limma package of Bioconductor [31]. Data preprocessing, including background correction, normalization, and expression set summaries, was performed using RMA. Array quality was assessed using arrayQualityMetrics package of Bioconductor [32]. Estimated fold change for each comparison was calculated using ANOVA, and resulting *t*-statistics from each comparison were modified using an intensity-based empirical Bayes method (IBMT) [33]. Genes with *P* value < 0.05, absolute fold change > 1.2, and average intensity > 100 fluorescent units were considered significantly differentially expressed. These cutoffs are consistent with similar studies in the literature [34–37]. Significant genes were further analyzed by Fisher's exact tests to identify overrepresented gene ontology categories and KEGG pathways. For these categories and pathways, a false discovery rate (FDR) below 0.05 and an odds ratio (log OR) outside the interval 0.67 to 1.5 were considered significant. To eliminate GO categories that are too broad to be informative, we focused on categories of fewer than 100 genes.

### 2.5. PCR Array and Real-Time RT-PCR

The microarray results for untreated and MDMA-treated rats were validated with the rat heat shock protein and chaperone PCR array (SA Biosciences), which profiles the expression of 84 Heat Shock Protein genes that regulate protein folding. The array contains a set of optimized real-time PCR primer assays on a 96-well plate for pathway focused genes as well as appropriate RNA quality controls. It performs gene expression analysis with real-time PCR sensitivity and the multigene profiling capability of a microarray. The assay was run and evaluated at the SA Biosciences PCR Array Service.

For Per2, we measured the gene expression levels by real-time RT-PCR, using primers 5′-CTTCTGGTCTGGACTGCACA-3′ (forward) and 5′-TGAGTCTGAGGTGGCAGATG-3′ (reverse), 1.5 mM magnesium, and 55°C annealing temperature for 40 cycles. Actin served as a loading control, and a reference cDNA validated each run.

## 3. Results

Exposure to chronic, unpredictable stress led to an overall moderate genetic response that comprised increases and decreases in gene expression levels. The consecutive administration of MDMA dramatically altered the profile of stress-responsive genes as follows.

**Figure 1:**
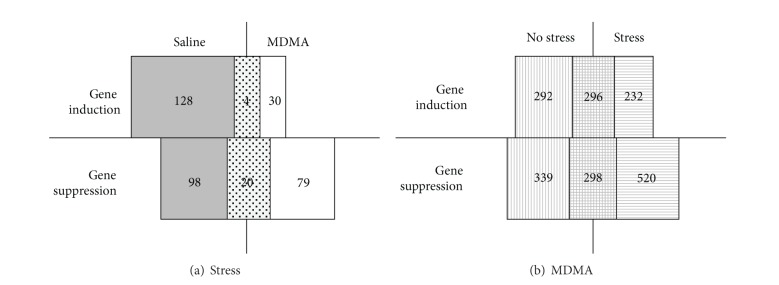
Gene expression changes following exposure to stress and MDMA. (a) Relative number of genes induced or suppressed by stress in the hippocampus of rats subsequently treated with saline or MDMA. Boxes outlined above the horizontal line indicate genes induced by stress and boxes outlined below the horizontal line indicate the number of genes suppressed by stress. Boxes outlined to the left and right of the vertical line indicated the number of genes up- or downregulated by saline or MDMA treatment, respectively. The shaded boxes in the middle of the diagram indicate the number of genes induced or suppressed by stress that were identical in saline and MDMA-treated animals and are considered MDMA-resistant. (b) Relative number of genes induced or suppressed by MDMA in the rat hippocampus of rats previously exposed to no stress or chronic unpredictable stress.

- 118 genes were suppressed by stress alone. Most of these (i.e., 98) were distinct from the 99 genes suppressed by stress with ensuing exposure to MDMA (Figure 1(a)). Remarkably, there were 20 genes suppressed by stress in a fashion that was resistant to the consecutive exposure to MDMA (Table 1).
- 132 genes were upregulated by stress alone. Most (i.e., 128) of these genes differed from the only 34 genes upregulated after stress with ensuing administration to MDMA (see Figure 1(a)). The significance of the four genes (hypothetical protein LOC689399, hypothetical protein LOC680454, and hypothetical protein LOC686031, similar to 60S ribosomal protein L7a) induced by stress in a MDMA-resistant manner is unclear as all of them encode hypothetical or yet uncharacterized proteins.
- Exposure to chronic stress resulted in altered gene expression in the KEGG pathway rno04710 (circadian rhythm). Four of twelve genes in this group were suppressed by stress, leading to a Fisher FDR of 3.23 × 10^−02^ and a log odds ratio of 2.95. However, the stress-induced alteration in gene expression in the circadian rhythm pathway was not evident in stressed rats treated subsequently with MDMA (Table 2). For one of the affected genes, Per2, the stress-dependent downregulation and MDMA-mediated reversal thereof was confirmed by real-time RT-PCR (not shown).

Exposure to MDMA led to the altered expression of 1225 genes in the hippocampus, with 588 genes being upregulated and 637 genes being suppressed (Figure 1(b)). Four gene ontology categories were affected (Table 3), comprising unfolded protein binding, protein folding, calmodulin-dependent protein kinase activity, and neuropeptide signaling pathway. The impact on protein folding seems to be of particular importance in the genetic response to MDMA as evidenced by the differential regulation of a large number of heat shock protein genes, for example, Hsp40, Hspd1, Hspa8, and Hsp90ab1 (Table 4(a)). The MDMA effect on heat shock proteins was validated by real-time RT-PCR array on samples from unstressed MDMA-treated versus unstressed untreated rats, which confirmed the MDMA-induced upregulation of several heat shock proteins and chaperones. In particular, Cct5, Dnajb1, Dnaja4, Dnajb11, Dnajb4, Hsp90ab1, Cct6a, SerpinH1, and Dnaja2 were increased under MDMA treatment in both microarray analysis and real-time RT-PCR (Table 4(b)). A moderate induction (1.2- to 1.4-fold) was seen in the PCR array in Cct3, Tcp1, and Cct4 (not shown), which were comparably moderately increased in the microarray.

According to gene ontology analysis, the genetic response by unstressed rats to MDMA (described in the preceding paragraph) was different from the genetic response by stressed rats to MDMA, indicating that MDMA induces altered responses to tissue damage in nerve sheaths and altered sensory processing in stressed, but not unstressed individuals as follows.

- When compared to stressed rats not treated with MDMA, the administration of MDMA following stress downregulated the related categories wound healing (FDR 1.30 × 10^−5^, log OR 1.626), tissue regeneration (FDR 1.76 × 10^−3^, log OR 1.761), myeloid leukocyte activation (FDR 2.30 × 10^−2^, log OR 2.03), and cytokine-cytokine receptor interaction (FDR 3.01 × 10^−2^, log OR 1.638). Further, stress plus MDMA versus stress alone suppressed ensheathment of neurons (FDR 2.47 × 10^−3^, log OR 1.595), axon ensheathment (FDR 2.47 × 10^−3^, log OR 1.595), myelination (FDR 5.33 × 10^−3^, log OR 1.556), and myelin sheath (8.17 × 10^−3^, log OR 2.436). This suggests that tissue damage conferred by MDMA on the stressed hippocampus was exerted particularly to the axon sheaths. MDMA had no effect on these gene ontology categories when given to unstressed rats.
- When compared to stressed rats treated with saline, administration of MDMA following stress upregulated the related categories rhodopsin-like receptor activity (FDR 1.54 × 10^−4^, log OR 1.733), sensory perception of chemical stimulus (FDR 3.22 × 10^−3^, log OR 2.537), neurotransmitter binding (FDR 8.49 × 10^−3^, log OR 1.807), neurotransmitter receptor activity (FDR 8.49 × 10^−3^, log OR 1.962), sensory perception of smell (FDR 1.89 × 10^−2^, log OR 2.469), response to protein stimulus (FDR 2.11 × 10^−2^, log OR 1.894), and detection of stimulus involved in sensory perception (FDR 3.59 × 10^−2^, log OR 2.246). This implies sensory changes exerted by MDMA on the stressed, but not the unstressed hippocampus.

## 4. Discussion

The present study examined the effects of chronic stress and subsequent MDMA treatment on gene transcription in the rat hippocampus. The key findings are the following: (1) MDMA alone increases the expression of genes in several gene ontology categories, including protein folding and unfolded protein binding, (2) MDMA upregulates the genes for several heat shock proteins, (3) MDMA negatively affects the expression of genes related to axon sheaths and tissue remodeling in stressed rats, (4) chronic stress alters gene expression within the circadian rhythm pathway, and this effect is absent in rats treated subsequently with MDMA, and (5) the number of stress-responsive genes is lessened by MDMA treatment.

The genetic responses to repeated stress included over two hundred genes that were either up- or downregulated. The reduction in the number of these genes by consecutive MDMA administration implies the potential for a drug-induced amelioration of the stress response. Among the genetic adaptations to stress, transcripts in the KEGG pathway for circadian rhythm were downregulated in response to chronic, unpredictable stress. One prominent clinical characteristic of stress disorder, particularly PTSD, in humans is abnormalities in the circadian rhythms of several physiological responses such as cortisol, blood pressure, and sleep [38–40]. In the present study, the suppressive effect of stress on gene transcription within the KEGG pathway for circadian rhythm was not manifest in stressed rats treated subsequently with MDMA. There is evidence that MDMA may affect sleep cycles [41], implying that it could have the potential to reverse or reduce stress-induced sleep disturbance. MDMA has been shown to be beneficial in treating the symptoms of PTSD [2]. While it is not yet known whether this clinical response is related to a restoration of circadian rhythms, the anxiolytic drug prazosin can reduce stress-induced sleep disturbances [42], providing a rationale for non-antidepressant restoration of the circadian rhythm. In general, MDMA is under discussion for the treatment of anxiety disorders [43]. This drug significantly altered the pattern of stress-induced gene induction and suppression. It remains to be determined whether the ability of MDMA to alter the genetic response to stress is of significance in the clinical response of PTSD patients to MDMA.

One component of the psychopharmacology of MDMA is that human abusers of the drug report enhanced sensory perceptions. In the present study, MDMA administration to chronically stressed rats exhibited an upregulation of related gene categories associated with sensory perception when compared to stressed rats not given MDMA. These findings suggest that MDMA may exert persistent effects on sensory perception that outlast the acute effects of the drug on neurotransmitter release.

Exposure to prior chronic stress impacted the genetic responses to MDMA. Notably, gene categories of tissue regeneration, axon ensheathment, myelin sheath, and cytokine-cytokine receptor interaction were significantly downregulated following MDMA treatment to rats exposed to chronic stress when compared to unstressed rats given MDMA or to stressed rats not given MDMA. Hence, chronic stress appears to augment the deleterious effects of MDMA on the neuronal structure. Callahan et al. [44] have concluded that MDMA produces structural damage to axonal transport mechanisms in multiple brain regions. The susceptibility to that damage or the intensity of the damage may be enhanced consecutively to stressing experiences. Although the identity of neurons affected by the combination of stress and MDMA is unknown, it is of interest that chronic, unpredictable stress augments the persistent deficits in serotonergic neurons produced by MDMA [30].

The gene expression changes observed in this study are consistent with the existing literature. The MDMA-induced alterations in gene ontology categories are consistent with transcriptional events associated with drug-induced neuronal damage. Genes related to protein folding and unfolded protein binding were upregulated 24 hours following MDMA treatment. Gene transcripts for heat shock proteins were notably upregulated following MDMA administration. This finding is consistent with previous reports that MDMA increases heat shock protein mRNAs in the mouse striatum [16] and heat shock protein levels in several brain regions [45–47]. The effects of MDMA on heat shock protein gene expression are viewed as a response to cellular stress that may accompany MDMA-induced hyperthermia and/or oxidative stress.

Treatment with MDMA also increased gene expression in GO categories related to cell signaling (e.g., neuropeptide signaling pathway and calmodulin-dependent protein kinase activity). This finding is in accord with the reports of Thiriet et al. [48] and Salzmann et al. [16] in which MDMA was shown to increase transcription for several proteins belonging to signal transduction pathways. These changes can be viewed, most likely, as a consequence of 5-HT receptor activity subsequent to the MDMA-induced increase in extracellular 5-HT in the hippocampus [49].

The use of psychedelic drugs to treat emotional disorders has been controversial due to the intrinsic neurotoxicity of these agents [1]. The evaluation of such therapies largely depends, in the short term, on cognitive and psychological evaluations that may be particularly susceptible to placebo effects or interpretive differences. The supplementation of clinical studies with ex vivo molecular assessments may aid in providing mechanistic insights. The present investigation of hippocampal gene expression profiles has elucidated the potential of MDMA to ameliorate the genetic response to chronic unpredictable stress and to reverse stress-induced sleep disorders. However, it has also found evidence that the toxicity of MDMA on nerve sheaths may be increased in individuals previously exposed to stressful experiences.

## Figures and Tables

**Table 1:** Stress suppressed genes that are resistant to consecutive MDMA exposure.

Entrez ID	Symbol	Gene name		No MDMA		MDMA	
			Average intensity	−Fold change	*P* value	−Fold change	*P* value
361619	MGC72973	Beta-glo	744.46	−1.62	0.02	−1.58	0.01
287167	LOC287167	Globin, alpha	512.31	−1.61	0.02	−1.29	0.04
24440	Hbb	Hemoglobin, beta	1327.73	−1.60	0.02	−1.55	0.00
499947	Cbln4	Cerebellin 4 precursor	103.48	−1.51	0.04	−1.78	0.00
29139	Dcn	Decorin	203.33	−1.45	0.04	−1.43	0.00
300850	Gsta4	Glutathione S-transferase, alpha 4	112.68	−1.41	0.03	−1.49	0.01
362489	Runx1t1	Runt-related transcription factor 1; translocated to, 1	152.25	−1.36	0.04	−1.22	0.00
313729	Errfi1	ERBB receptor feedback inhibitor 1	725.49	−1.35	0.00	1.21	0.00
252917	Nr1d1	Nuclear receptor subfamily 1, group D, member 1	347.48	−1.34	0.00	−1.45	0.00
685671	LOC685671	Similar to myocyte enhancer factor 2C	1233.46	−1.31	0.02	−1.34	0.00
24330	Egr1	Early growth response 1	286.86	−1.31	0.02	−1.26	0.03
289440	Abhd7	Abhydrolase domain containing 7	125.15	−1.30	0.02	−1.26	0.00
308831	Odz4	Odd Oz/ten-m homolog 4 (Drosophila)	162.69	−1.26	0.01	−1.25	0.00
316351	Npas2	Neuronal PAS domain protein 2	255.91	−1.25	0.01	−1.24	0.00
114858	Shc3	Src homology 2 domain-containing transforming protein C3	301.74	−1.24	0.00	−1.26	0.00
310358	RGD1308448	Similar to RIKEN cDNA B130016O10 gene	169.03	−1.23	0.03	−1.22	0.00
294250	Bat2	HLA-B associated transcript 2	266.79	−1.22	0.01	−1.22	0.00
24309	Dbp	D site albumin promoter binding protein	961.48	−1.21	0.00	−1.28	0.00
291356	RGD1563437	Similar to KIAA1217	116.04	−1.21	0.04	−1.22	0.00
116470	Stx1a	Syntaxin 1A (brain)	436.23	−1.20	0.04	−1.27	0.00

Stress suppressed genes that are resistant to consecutive MDMA exposure. The average intensity of all genes shown is above threshold. All −fold changes and *P* values are significantly differentially expressed as defined in Section 2.

**Table 2:** KEGG category rno04710 (circadian rhythm).

Entrez ID	Symbol	Gene Name		Stress versus no stress			
				No MDMA		MDMA	
			Average intensity	−Fold change	*P* value	−Fold change	*P* value
29657	Arntl	Aryl hydrocarbon receptor nuclear translocator-like	135.80	−1.07	0.32	−1.08	0.12
63840	*Per2 *	*Period homolog 2 (Drosophila) *	108.53	−**1.21**	**0.01**	−1.12	0.14
79431	Bhlhb2	Basic helix-loop-helix domain containing B2	638.68	−1.03	0.73	−1.07	0.15
299691	Cry1	Cryptochrome 1 (photolyase-like)	116.72	1.12	0.15	1.12	**0.03**
64462	Csnk1d	Casein kinase 1, delta	271.06	−1.05	0.37	−1.03	0.38
58822	Csnk1e	Casein kinase 1, epsilon	382.89	−1.02	0.74	−1.09	**0.02**
287422	*Per1 *	*Period homolog 1 (Drosophila) *	165.79	−**1.34**	**0.00**	1.00	0.91
170917	Cry2	Cryptochrome 2 (photolyase-like)	540.10	−1.09	0.10	−1.13	**0.01**
60447	Clock	Circadian locomoter output cycles kaput	244.29	−1.14	**0.02**	1.01	0.84
252917	*Nr1d1 *	*Nuclear receptor 1D1 *	347.48	−**1.34**	**0.00**	−**1.45**	**0.00**
316351	*Npas2 *	*Neuronal PAS domain protein 2 *	255.91	−**1.25**	**0.01**	−**1.24**	**0.00**
78962	Per3	Period homolog 3 (Drosophila)	115.64	−1.10	0.25	−1.05	0.35

The average intensity of all genes shown is above threshold. Fold changes above or below threshold and significant *P* values are shown in bold. On this basis, the italicized genes are judged to be suppressed by stress.

**Table 3:** Gene ontology analysis of MDMA-induced genes in the hippocampus.

Category type	Category ID	Category description	No stress			
			No MDMA versus MDMA			
			Genes in category	All genes in category	Fisher FDR	logOR
GO	GO:0051082	Unfolded protein binding	21.00	63.00	0.00	2.00
GO	GO:0006457	Protein folding	25.00	94.00	0.00	1.68
GO	GO:0004683	Calmodulin-dependent protein kinase activity	7.00	14.00	0.01	2.65
GO	GO:0007218	Neuropeptide signaling pathway	7.00	19.00	0.04	2.16

All rats were unstressed. FDR: false discovery rate. logOR: logarithm of the odds ratio.

**Table:** (a)

Entrez ID	Symbol	Gene name	Average intensity	Stress versus no stress				MDMA versus no MDMA			
				No MDMA		MDMA		No stress		Stress	
				−Fold change	*P* value	−Fold change	*P* value	−Fold change	*P* value	−Fold change	*P* value
361384	Dnajb1	DnaJ (Hsp40) homolog, subfamily B, member 1	363.16	1.02	0.81	−1.09	0.50	**2.18**	**0.00**	**1.96**	**0.00**
65028	Dnaja1	DnaJ (Hsp40) homolog, subfamily A, member 1	1222.76	1.04	0.43	1.03	0.51	**1.45**	**0.00**	**1.44**	**0.00**
295549	Dnajb4	DnaJ (Hsp40) homolog, subfamily B, member 4	668.67	1.02	0.73	1.01	0.87	**1.42**	**0.00**	**1.40**	**0.00**
300721	Dnaja4	DnaJ (Hsp40) homolog, subfamily A, member 4	232.20	1.07	0.48	−1.10	0.21	**1.39**	**0.01**	1.18	**0.01**
288620	Cct6a	chaperonin subunit 6a (zeta)	395.91	1.03	0.73	−1.02	0.50	**1.37**	**0.00**	**1.30**	**0.00**
360734	Dnajb11	DnaJ (Hsp40) homolog, subfamily B, member 11	472.49	1.01	0.93	−1.02	0.75	**1.36**	**0.00**	**1.33**	**0.00**
29345	Serpinh1	Serine (or cysteine) peptidase inhibitor, clade H, member 1	469.00	−1.04	0.77	−1.12	0.47	1.36	0.15	1.26	**0.00**
295230	Cct3	chaperonin subunit 3 (gamma)	686.33	1.03	0.62	−1.06	0.26	**1.35**	**0.00**	**1.24**	**0.00**
311456	Mkks	McKusick-Kaufman syndrome protein	320.40	1.06	0.33	1.02	0.81	**1.35**	**0.00**	**1.30**	**0.00**
64202	Calr	Calreticulin	1882.72	1.03	0.63	−1.05	0.29	**1.32**	**0.00**	**1.22**	**0.00**
63868	Hspd1	Heat shock protein 1 (chaperonin)	1509.64	1.01	0.83	−1.00	0.98	**1.28**	**0.01**	**1.26**	**0.00**
29374	Cct4	Chaperonin subunit 4 (delta)	662.36	1.03	0.63	−1.04	0.45	**1.28**	**0.00**	1.20	**0.00**
25719	Scg5	Secretogranin V	2031.48	1.05	0.34	−1.00	0.94	**1.26**	**0.00**	1.19	**0.00**
362862	Tra1	Tumor rejection antigen gp96	2797.04	1.03	0.63	1.04	0.33	**1.25**	**0.01**	**1.26**	**0.00**
24818	Tcp1	t-complex protein 1	474.45	1.02	0.80	−1.01	0.81	**1.24**	**0.00**	**1.20**	**0.01**
301252	Hsp90ab1	Heat shock protein 90 kDa alpha (cytosolic), class B member 1	4755.12	1.01	0.72	−1.03	0.35	**1.24**	**0.00**	1.18	**0.00**
24468	Hspa8	Heat shock protein 8	3661.99	1.02	0.84	1.06	0.33	**1.22**	**0.03**	**1.27**	**0.00**
294864	Cct5	Chaperonin subunit 5 (epsilon)	1223.73	1.04	0.45	1.01	0.82	**1.22**	**0.00**	1.18	**0.00**
368044	Atp6v1g2	ATPase, H+ transporting, V1 subunit G isoform 2	1494.80	1.02	0.72	−1.03	0.38	**1.22**	**0.00**	1.15	**0.00**
294236	Gtf2h4	General transcription factor II H, polypeptide 4	106.29	1.08	0.39	−1.04	0.36	**1.21**	**0.01**	1.08	0.22
84026	Dnaja2	DnaJ (Hsp40) homolog, subfamily A, member 2	1040.62	1.05	0.47	1.06	0.06	**1.21**	**0.00**	**1.23**	**0.00**
619393	Dnajc12	DnaJ (Hsp40) homolog, subfamily C, member 12	367.66	1.06	0.29	−1.01	0.72	**1.21**	**0.00**	**1.12**	**0.03**
89811	Vegfb	Vascular endothelial growth factor B	330.14	−1.03	0.66	−1.04	0.33	−**1.22**	**0.01**	−**1.23**	**0.00**
299313	Uxt	Ubiquitously expressed transcript	616.75	−1.05	0.51	1.04	0.20	−**1.29**	**0.00**	−1.19	**0.00**
29427	Aif1	Allograft inflammatory factor 1	172.49	−1.07	0.47	−**1.25**	**0.00**	−**1.39**	**0.00**	−**1.62**	**0.00**

Individual members of the gene ontology category Unfolded Binding Protein are shown. The average intensity of all genes shown is above threshold. Fold changes and *P* values beyond the cutoff for significance are in bold. For clarity, genes that do not reach significance in any category are not shown. Genes induced by MDMA are separated by a free line.

**Table:** (b)

Gene symbol	Fold regulation
Hspa5	2.6069
Cct5	2.5859
Dnajb1	2.3823
Cryab	2.3305
Hspb1	2.2668
Hspa2	2.215
Bag3	2.0596
Dnaja4	1.8908
Cct7	1.8412
Dnajb11	1.8159
Hspa14	1.8117
Dnajb4	1.7724
Ldha	1.7399
Dnaja1	1.6982
Hsph1	1.6806
Dnajc7	1.6537
Hsp90ab1	1.5955
Cct6a	1.5808
Hsp90aa1	1.5609
Cct2	1.5112
Hsp90b1	1.5025
Serpinh1	1.4973
Dnaja2	1.4346
Hspa4l	1.4198

Real-time PCR validation of chaperone and heat shock gene upregulation by MDMA exposure (2 rats) versus unstressed untreated rats (3 rats). Fold change (2^−ΔΔCt^) is the normalized gene expression (2^−ΔCt^) in the test sample divided by the normalized gene expression (2^−ΔCt^) in the control sample. Fold regulation represents fold change results in a biologically meaningful way. Fold change values greater than one indicate a negative or downregulation and the fold regulation is the negative inverse of the fold change. Shown are positive changes in fold regulation by MDMA of at least 1.4 over controls.
